# Between endothelial activation and stress index (EASIX) and mortality in acute respiratory distress syndrome (ARDS) patients: a multicenter retrospective study

**DOI:** 10.3389/fphys.2025.1570988

**Published:** 2025-09-26

**Authors:** Juan Chen, Jing Lv, Meijun Liu, Xue Dai, Wang Deng

**Affiliations:** ^1^ Department of Pulmonary and Critical Care Medicine, Second Affiliated Hospital of Chongqing Medical University, Chongqing, China; ^2^ The First batch of key Disciplines on Public Health in Chongqing, Chongqing, China

**Keywords:** acute respiratory distress syndrome (ARDS), endothelial activation and stress index (EASIX), intensive care units (ICU), mortality, A multicenter retrospective study

## Abstract

**Background:**

The Endothelial Activation and Stress Index (EASIX), calculated as [lactate dehydrogenase (U/L) × creatinine (mg/dL)] ÷ platelet count (109/L), serves as a reliable biomarker for endothelial dysfunction. Endothelial damage significantly contributes to the pathophysiological mechanisms underlying acute respiratory distress syndrome (ARDS). However, the relationship between EASIX and ARDS patients remains to be fully elucidated.

**Methods:**

To evaluate the relationship between EASIX and outcomes in patients with acute respiratory distress syndrome (ARDS), in two cohorts we used Cox proportional hazards models and applied restricted cubic spline methods. Then we stratify EASIX into higher Log2_EASIX and lower Log2_EASIX groups, matched baseline data from the two stratified groups in both cohorts using propensity score matching to reduce confounding bias. Additionally, we further analyzed the differences in clinical outcomes between the higher Log2_EASIX and lower Log2_EASIX groups and performed Kaplan-Meier analysis on the matched data.

**Results:**

In the MIMIC-IV cohort, compared to the survival group, within the 28 days, the non-survival group had higher Log2_EASIX (Survival: Non-survival = 1.35 [0.16, 2.80]: 2.08 [0.79, 3.59], P = 0.002),and in the CQMU cohort, the non-survival group had higher Log2_EASIX (Survival: Non-survival = 1.91 [1.48, 2.43]: 2.34 [1.89, 3.01], P < 0.0001), Even after adjusting for potential confounders, individuals exhibiting elevated Log2_EASIX values still faced a greater risk of mortality during hospitalization and at 28-, 60-and 90-day post-admission.

**Conclusion:**

Elevated EASIX levels were found to be positively correlated with a higher risk of mortality in patients with ARDS. Assessing EASIX levels could be a promising biomarker for predicting overall survival in ARDS.

## Introduction

Acute respiratory distress syndrome (ARDS), one of the most prevalent severe respiratory diseases, has a mortality rate as high as 40% (Pisani et al., 2021). ARDS arises from injury to the endothelial cells (ECs) of pulmonary capillaries and the epithelial cells of the alveoli, triggered by significant insults such as infections, trauma, and burns. This damage leads to widespread pulmonary interstitial and alveolar edema, ultimately resulting in persistent hypoxemia and respiratory distress (Bos and Ware, 2022). The barrier between alveoli and capillaries is comprised of a delicate layer formed by alveolar epithelial cells and capillary ECs. Injury to this barrier is a hallmark of ARDS, leading to distinct physiological disruptions (Matthay et al., 2019), including pulmonary edema, surfactant dysfunction, and impaired gas exchange (Huppert et al., 2019). The endothelium functions as a multifaceted organ that regulates vascular tone, promotes angiogenesis, manages coagulation, and facilitates the recruitment of leukocytes and platelets (Bailey et al., 2023). It plays a critical role in the pathogenesis of ARDS. Studies have confirmed that decitabine improves the prognosis of elderly patients with ARDS by activating endothelial regeneration and repair (Huang et al., 2023). In a mouse model of Acute Lung Injury (ALI), increasing levels of MicroRNA-1 (miR-1) has been shown to have a protective effect on endothelial cells (ECs), and overexpression of miR-1 in lung ECs can enhance the survival rate of mice with pneumonia-induced ALI (Korde et al., 2023). Therefore, targeted therapy of the endothelium may represent a novel approach to enriching ARDS treatment. Despite recent advancements in understanding the pathogenesis and treatment of ARDS, there remains a paucity of highly sensitive and specific biomarkers that can serve as diagnostic tools in clinical practice (Gorman et al., 2022). Bos et al. suggested that indicators of endothelial or epithelial damage, along with the systemic or alveolar responses of the host, may serve as potential biomarkers for ARDS diagnosis, the pulmonary vessel endothelium represents a critical site of cellular dysfunction in patients with ARDS (Bos et al., 2022). Early manifestations include increased capillary leakage, while late-stage features often involve EC death (Bachofen and Weibel, 1982). Studies have demonstrated that damage to the endothelial barrier in sepsis-induced ALI and ARDS is associated with the activation of ECs and their interactions with immune and stromal cells (Deng et al., 2023). Furthermore, severe endothelial dysfunction can lead to hemostatic disorders, abnormal vascular reactivity, and tissue edema (Qiao et al., 2024). Endothelial damage activates pro-coagulation mechanisms, which may result in increased microvascular thrombosis within the lungs and dead space (Nuckton et al., 2002). This microvascular thrombosis, combined with substantial damage to the microvascular network, can contribute to pulmonary hypertension and acute right heart failure, thereby affecting the prognosis of ARDS patients (Petit et al., 2021).

The endothelial activation and stress index (EASIX) is a score that serves as a biomarker associated with endothelial dysfunction and can be easily derived from standard laboratory indicators (Luft et al., 2017). This index is recognized as a reliable measure of endothelial risk in both immune-mediated and malignant conditions (Luft et al., 2021) and is utilized to predict endothelial-related complications and mortality following allogeneic stem cell transplantation and acute graft-versus-host disease (GvHD) (Luft et al., 2017; Varma et al., 2020; Korell et al., 2022; Luft et al., 2020). Furthermore, EASIX has been shown to correlate with mortality in individuals diagnosed with low-risk myelodysplastic syndromes and multiple myeloma (Merz et al., 2019; Song et al., 2020). Beyond hematological diseases, EASIX has recently been applied to various conditions associated with endothelial damage. For instance, in patients with novel coronavirus pneumonia, EASIX is closely related to patient prognosis. Studies indicate that approximately 50% of patients with an EASIX score of ≥2 upon admission experience a severe disease course (either death or the necessity for mechanical ventilation) (Luft et al., 2021). Additionally, in patients with sepsis, an elevated EASIX correlates with a significantly higher risk of all-cause mortality at both 28 days and 90 days (Xu et al., 2023). Considering that the pulmonary capillary endothelium is a critical site of cellular dysfunction in patients with ARDS, this study aimed to investigate the relationship between the EASIX and overall mortality rates in this patient population. We hypothesized that elevated levels of EASIX are associated with an increased risk of mortality among individuals diagnosed with ARDS.

## Methods

### Data sources

This study is an international, multicenter retrospective study conducted using data from two cohorts. The MIMIC-IV cohort was from the Medical Information Mart for Intensive Care (MIMIC-IV) database, and the CQMU cohort was from the Second Affiliated Hospital of Chongqing Medical University (CQMU). The MIMIC-IV is a longitudinal, single-center database that includes 247,366 individuals and 196,527 adults who visited Beth Israel Deaconess Medical Center from 2008 to 2019 (Johnson et al., 2023). The Second Affiliated Hospital of Chongqing Medical University (CQMU) is a national tertiary-level hospital in China that integrates medical care, teaching, scientific research, and preventive healthcare. It treats a large number of critically ill patients every year.

The study adhered to the STROCSS reporting guidelines. We have received authorization to utilize the MIMIC-IV database, as one of our team members (record ID 12892548) has officially registered as a certified user of PhysioNet. Following the completion of the Human Subjects Research Training Course and the signing of the Data Use Agreement (DUA), the Beth Israel Deaconess Medical Center (Boston, MA) Institutional Review Board granted approval for the use of this dataset; therefore, the requirement for informed consent was waived. The CQMU cohort study followed procedures that were in accordance with ethical standards set by the Chongqing Medical University Ethics Committee (registration number 2022729). This work obtained separate, written consent from ARDS patients in this study.

### Study population

Adult patients with ARDS were included in our study. Due to the retrospective nature of this study and the fact that patient data were gathered long ago, the criteria for including patients with ARDS adhered to the classic diagnostic standards, the Berlin criteria (Ranieri et al., 2012). Our inclusion criteria were as follows: (1) Onset timing: emergence or deterioration of respiratory symptoms (e.g., dyspnea, shortness of breath) within 1 week among high-risk individuals; (2) Imaging of the chest: the presence of patchy opacities in both lungs that cannot be thoroughly accounted for by pleural effusion, atelectasis, or nodular formations; (3) Causes of pulmonary edema: respiratory failure not fully attributable to heart failure or fluid overload, necessitating the exclusion of risk factors that require objective evaluations (like echocardiography) to distinguish between cardiogenic and non-cardiogenic pulmonary edema. (4) Oxygenation: mild: 200 mmHg < oxygenation index (ratio of arterial oxygen partial pressure to inspired oxygen concentration) ≤ 300 mmHg (1 mmHg = 0.133 kPa), and positive end-expiratory pressure (PEEP) or continuous positive airway pressure ventilation pressure ≥5 cmH2O (1 cmH2O = 0.098 kPa); moderate: 100 mmHg < oxygenation index≤ 200 mmHg, and PEEP ≥5 cmH2O; severe: oxygenation index≤ 100 mmHg and PEEP ≥5 cmH2O. Our exclusion criteria are as follows: (a) no admissions to the ICU or multiple admissions; (b) incomplete data for creatinine, lactate dehydrogenase, or platelet counts within the first 24 h of ICU admission; (c) age under 18 years; (d) an ICU stay of less than 24 h; (e) individuals for whom the 90-day survival status could not be determined.

### Extraction of variables

Variables were obtained from the MIMIC IV database using PostgreSQL (version 9.6). The data included demographic information such as age and sex, as well as laboratory test results recorded within 24 h of ICU admission. These results encompassed white blood cells (WBC), platelets (PLT), hemoglobin (Hb), blood creatinine, blood urea nitrogen (BUN), prothrombin time (PT), partial thromboplastin time (PTT), international normalized ratio (INR), lactate dehydrogenase (LDH), alanine aminotransferase (ALT), aspartate aminotransferase (AST), and the ratio of arterial oxygen partial pressure to inspired oxygen concentration (P/F). Additionally, sequential organ failure assessment (SOFA) scores and treatment parameters, including the use or non-use of renal replacement therapy (RRT) and invasive ventilation or vasopressor drugs (such as norepinephrine, epinephrine, dobutamine, phenylephrine, vasopressin, dopamine, and milrinone), were collected. Comorbidity data included hypertension, chronic pulmonary disease, rheumatic disease, diabetes, renal failure, and malignant cancer. In our study, EASIX was calculated using the formula: [LDH (U/L) × creatinine (mg/dL)] ÷ platelet count (10^9/L).

### Management of missing data and statistical analysis

The analysis disregarded variables that had over 20% missing values to lessen potential bias; the missing data rate for all other variables was below 10%, and the mean or median was imputed for missing values using SPSS software (version 27).

Prior to the analysis, EASIX values underwent log2 transformation because they were skewedly distributed (Xu et al., 2023). Regarding data presentation, continuous variables were presented as the mean and standard deviation (SD) for normally distributed data or the median and interquartile range for skewedly distributed data. Conversely, categorical variables were expressed as frequencies and percentages. Continuous variables were compared applying the Kruskal–Wallis test. For categorical variables, the χ2 test and Fisher’s exact test were utilized to ensure the accuracy and validity of the comparisons, respectively.

The survival time was analyzed using both univariate and multivariate approaches with the Cox regression model. Additionally, the association between Log2_EASIX and mortality over a 28-day period was analyzed using the Cox proportional hazards model. To ensure the validity of the results, both unadjusted and multivariate-adjusted models were employed. The adjustment covariates were selected based on their clinical relevance and the results from the multivariate COX regression analysis. In the first model, adjustments were made for age and gender. The second model included further adjustments for the comorbidities (Hypertension, Chronic pulmonary disease, Diabetes, Renal failure, and Malignant cancer). The third model additionally accounted for Los_ ICU (ICU length of stay (day)). Then, in the fourth model, additional adjustments were made for treatments of intervention (vasopressor, renal replacement treatment and invasive mechanical ventilation). Lastly, in the fifth model, we further adjusted for laboratory indicators (hemoglobin, WBC, BUN, P/F(PaO2/FiO2), SOFA, INR, PT, PTT, ALT, AST).

In addition, the correlation between Log2_EASIX levels and 28-day all-cause mortality in ARDS patients was analyzed using RCS plots. The cutoff values obtained from the RCS curve were used to divide the data into high Log2_EASIX and low Log2_EASIX groups. Propensity score matching (PSM) was then performed separately in both cohorts to compare the baseline characteristics (age, gender, comorbidities) by creating matched cohorts, and standardized mean differences (SMD) were used to assess the matching quality, with an SMD >0.1 indicating potential imbalance (Zhang et al., 2019). After matching, balance was assessed using p-values, with P < 0.05 indicating significant imbalance. Finally, Kaplan-Meier survival curves were plotted for the two groups in the matched cohorts, and the survival curve comparison between the two groups was performed using the log-rank test. In the case of intersecting curves, the Tarone-Ware test was used.

All statistical analyses were conducted using the R package (version 2024.04.01) and IBM SPSS Statistics (version 27). Statistical significance was concluded for p values below 0.05 (two-tailed).

## Results

### Patient characteristics

**FIGURE 1:**
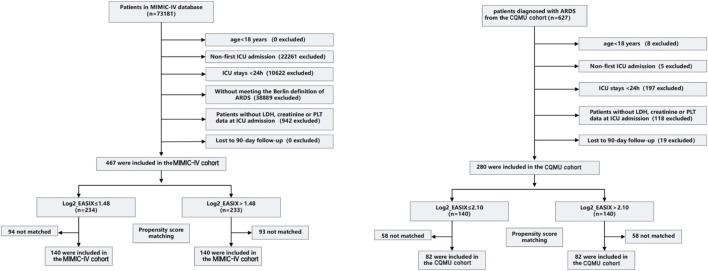
Enrollment Flowchart for the Selection of ARDS Patients. The MIMIC-IV cohort (cutoff value is 1.48) and the CQMU cohort (cutoff value is 2.10). Propensity score matching is used to compare baseline characteristics (including age, gender, and comorbidities) by creating matched cohorts. Standardized mean difference (SMD) > 0.1 suggests a potential imbalance, p-value <0.05 indicates a significant imbalance. ICU, intensive care unit; LDH, lactate dehydrogenase; PLT, platelet. ICU, intensive care unit; LDH, lactate dehydrogenase; PLT, platelet; EASIX, Endothelial Activation and Stress Index.

Out of 627 patients diagnosed with ARDS who were admitted to the ICU at the Second Affiliated Hospital of Chongqing Medical University between December 2021 and February 2024, 280 inpatients were selected for this study. Additionally, the MIMIC-IV database contributed 467 cases, representing a total of 73,181 ICU patients from 2008 to 2019. In both cohorts, cutoff values derived from the Restricted Cubic Spline (RCS) curve (the MIMIC-IV cohort: Log2_EASIX = 1.48; the CQMU cohort: Log2_EASIX = 2.10) were utilized to categorize the data into high Log2_EASIX and low Log2_EASIX groups. Following propensity score matching (PSM), the MIMIC-IV cohort comprised 140 individuals in both the high and low Log2_EASIX groups, while the CQMU cohort included 82 individuals in each group. Figure 1 illustrates the participant selection process (Figure 1).

The mean value of Log2_EASIX in the MIMIC-IV cohort was 1.48 [interquartile range (IQR): 0.41–2.98], while the mean value in the CQMU cohort was 2.10 (IQR: 1.68–2.70). The average lengths of ICU stay were 6.0 days for the MIMIC-IV cohort and 15 days for the CQMU cohort (p < 0.0001). Patients in the CQMU cohort exhibited higher levels of LDH and creatinine, longer ICU stays. Conversely, they had lower platelet counts and ratios of arterial oxygen partial pressure to inspired oxygen concentration (P/F) compared to those in the MIMIC-IV cohort. There was no significant difference in age between the two cohorts, both of which were predominantly male (MIMIC-IV cohort: CQMU cohort = 281 (60.2%): 202 (72.1%), P = 0.001). In terms of treatments, the MIMIC-IV cohort utilized more invasive mechanical ventilation, whereas the CQMU cohort employed more renal replacement therapy (RRT). No significant difference was observed in the use of vasopressor medications between the two cohorts. Regarding comorbidities, significant differences were noted between the cohorts for hypertension (p < 0.0001), chronic pulmonary disease (p = 0.023), and renal failure (p = 0.002; Table 1).

**TABLE 1:** Patient characteristics in the MIMIC-IV cohort and the CQMU cohort within 28days.

Variables	The MIMIC-IV cohort				The CQMU cohort				
	Total (n = 467)	Survival (n = 359)	Non-survival (n = 108)	P Value	Total (n = 280)	Survival (n = 143)	Non-survival (n = 137)	P Value	P-total
Age, M (Q1, Q3)	63.00 [51.00, 74.00]	63.00 [50.50, 74.00]	66.50 [54.00, 76.00]	0.056	66.00 [54.00, 75.00]	63.00 [50.00, 72.00]	70.00 [57.00, 77.00]	0.002	0.209
Gender (male)	281 (60.2%)	219 (61.0%)	62 (57.4%)	0.577	202 (72.1%)	104 (72.7%)	98 (71.5%)	0.929	0.001
Hypertension	88 (18.8%)	63 (17.5%)	25 (23.1%)	0.244	97 (34.6%)	46 (32.2%)	51 (37.2%)	0.445	<0.0001
Chronic pulmonary disease	155 (33.2%)	122 (34.0%)	33 (30.6%)	0.585	70 (25.0%)	30 (21.0%)	40 (29.2%)	0.147	0.023
Diabetes	123 (26.3%)	95 (26.5%)	28 (25.9%)	1.000	64 (22.9%)	38 (26.6%)	26 (19.0%)	0.170	0.329
Renal failure	93 (19.9%)	71 (19.8%)	22 (20.4%)	1.000	31 (11.1%)	13 (9.1%)	18 (13.1%)	0.374	0.002
Malignant cancer	77 (16.5%)	54 (15.0%)	23 (21.3%)	0.165	43 (15.4%)	20 (14.0%)	23 (16.8%)	0.628	0.761
Hemoglobin, M (Q1, Q3)	10.80 [9.20, 12.57]	10.80 [9.20, 12.50]	10.93 [9.20, 12.93]	0.738	11.40 [9.17, 13.50]	11.60 [9.30, 13.70]	11.30 [9.10, 13.10]	0.162	0.065
WBC, M (Q1, Q3)	12.35 [8.75, 17.62]	12.30 [8.68, 17.00]	13.00 [9.05, 18.14]	0.558	10.96 [7.32, 15.25]	10.79 [7.93, 14.80]	11.11 [7.01, 15.79]	0.410	0.006
BUN, M (Q1, Q3)	24.50 [16.00, 37.50]	23.50 [15.25, 37.75]	25.75 [18.50, 37.50]	0.116	9.11 [6.14, 14.42]	7.81 [5.54, 12.38]	10.37 [6.83, 15.86]	0.001	<0.0001
P/F, M (Q1, Q3)	198.00 [152.00, 242.50]	202.50 [154.00, 246.17]	178.00 [141.44, 227.25]	0.005	149.00 [110.75, 190.00]	160.00 [129.40, 201.00]	129.00 [101.00, 176.00]	<0.0001	<0.0001
SOFA, M (Q1, Q3)	9.00 [6.00, 12.00]	9.00 [6.00, 11.00]	10.50 [7.75, 13.25]	<0.0001	7.00 [4.00, 9.25]	6.00 [4.00, 9.00]	7.00 [5.00, 11.00]	0.006	<0.0001
INR, M (Q1, Q3)	1.30 [1.15, 1.70]	1.30 [1.15, 1.60]	1.55 [1.20, 2.10]	<0.0001	1.15 [1.04, 1.29]	1.13 [1.03, 1.25]	1.15 [1.06, 1.37]	0.038	<0.0001
PT, M (Q1, Q3)	14.50 [12.65, 18.40]	14.15 [12.60, 17.40]	16.60 [12.94, 22.10]	<0.0001	14.70 [13.70, 16.22]	14.60 [13.50, 15.80]	14.80 [13.90, 17.00]	0.026	0.206
APTT, M (Q1, Q3)	35.10 [29.38, 44.92]	33.40 [29.05, 42.50]	40.55 [31.60, 50.73]	0.001	17.40 [16.20, 18.80]	17.20 [15.90, 18.30]	17.60 [16.50, 19.30]	0.006	<0.0001
ALT, M (Q1, Q3)	39.00 [20.25, 117.75]	34.00 [19.75, 97.50]	50.50 [23.38, 193.12]	0.006	46.00 [28.00, 89.25]	41.00 [26.00, 70.00]	50.00 [30.00, 109.00]	0.028	0.069
AST, M (Q1, Q3)	66.00 [31.75, 212.50]	60.00 [29.75, 154.75]	89.00 [45.88, 375.25]	<0.0001	33.00 [19.00, 61.25]	33.00 [18.00, 56.00]	36.00 [20.00, 65.00]	0.253	<0.0001
platelets, M (Q1, Q3)	186.00 [119.00, 255.50]	185.00 [124.50, 256.50]	190.00 [100.00, 252.50]	0.590	169.00 [109.75, 239.25]	170.00 [118.50, 231.50]	163.00 [99.00, 244.00]	0.560	0.031
creatinine, M (Q1, Q3)	1.25 [0.85, 2.05]	1.20 [0.85, 2.00]	1.35 [0.99, 2.16]	0.212	1.91 [1.24, 2.70]	1.92 [1.34, 2.62]	1.84 [1.12, 2.76]	0.560	<0.0001
LDH, M (Q1, Q3)	346.00 [234.50, 532.75]	317.00 [228.75, 491.00]	460.75 [284.75, 843.75]	<0.0001	378.00 [284.75, 573.25]	332.00 [247.50, 476.00]	449.00 [329.00, 716.00]	<0.0001	0.013
Vasopressor	325 (69.6%)	234 (65.2%)	91 (84.3%)	<0.0001	177 (63.2%)	66 (46.2%)	111 (81.0%)	<0.0001	0.086
CRRT	75 (16.1%)	59 (16.4%)	16 (14.8%)	0.801	64 (22.9%)	32 (22.4%)	32 (23.4%)	0.958	0.027
IMV	387 (82.9%)	294 (81.9%)	93 (86.1%)	0.382	190 (67.9%)	75 (52.4%)	115 (83.9%)	<0.0001	<0.0001
Los_ ICU, M (Q1, Q3)	6.00 [3.00, 12.00]	7.00 [4.00, 12.00]	4.00 [3.00, 9.00]	<0.0001	15.00 [9.00, 23.25]	20.00 [13.00, 30.00]	10.00 [5.00, 16.00]	<0.0001	<0.0001
log2_EASIX, M (Q1, Q3)	1.48 [0.40, 2.98]	1.35 [0.16, 2.80]	2.08 [0.79, 3.59]	0.002	2.10 [1.69, 2.70]	1.91 [1.48, 2.43]	2.34 [1.89, 3.01]	<0.0001	<0.0001

Data are presented as the mean ± standard deviation (SD) for normal variables, median (IQR) for skewed variables, and numbers (proportions) for categorical variables. p-value <0.05 indicates a significant imbalance. Abbreviations:Los_ ICU, ICU, length of stay (day); SOFA, sequential organ failure assessment; EASIX, endothelial activation and stress index; Hb, hemoglobin (g/L); WBC, white blood cell (K/L); P/F, ratio of arterial oxygen partial pressure to inspired oxygen concentration; BUN, blood urea nitrogen (mg/dL); INR, international normalized ratio; PT, prothrombin time(s); APTT, activated partial thromboplastin time(s); ALT, Alanine aminotransferase (U/L); AST, Aspartate aminotransferase (U/L); LDH, Lactate dehydrogenase (U/L); CRRT, Continuous renal replacement therapy (day); IMV, Invasive mechanical ventilation (day).

### Outcomes

In the MIMIC-IV cohort comprising 467 individuals, 359 patients survived at 28 days, while 108 patients succumbed. Compared to the survival group, the Log2_EASIX in the mortality group was significantly higher (Survival: Non-survival = 1.35 [0.16, 2.80]: 2.08 [0.79, 3.59], P = 0.002). Additionally, the non-survival group exhibited elevated levels of SOFA, lactate dehydrogenase, INR, PT, APTT, ALT, and AST. Furthermore, a greater proportion of patients in this group received vasopressor treatment, and the oxygenation index was notably lower. No significant differences were observed between the survival and non-survival groups concerning gender, comorbidities (including hypertension, chronic pulmonary diseases, diabetes, renal insufficiency, and malignancies), invasive mechanical ventilation, or renal replacement therapy. In the CQMU cohort of 280 individuals, 143 survived at 28 days, whereas 137 died. Similar to the MIMIC-IV cohort, the Log2_EASIX in the mortality group was higher (Survival: Non-survival = 1.91 [1.48, 2.43]: 2.34 [1.89, 3.01], P < 0.0001). The non-survival group also demonstrated increased levels of BUN, SOFA, INR, PT, APTT, ALT, and LDH, along with a higher incidence of vasopressor treatment and mechanical ventilation. Additionally, the oxygenation index was lower in this group. In both cohorts, the mortality group exhibited higher Log2_EASIX values and shorter ICU stays, potentially reflecting the passive termination of treatment following patient death (Table 1).

**FIGURE 2:**
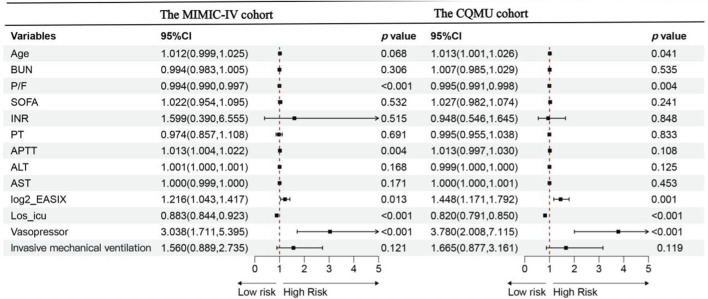
Forest plot of the multivariate Cox analysis for overall survival according to groups within 28 days for the MIMIC-IV cohort and CQMU cohort. Data are presented as median [interquartile range] or number (percentage).In the MIMIC-IV cohort: P/F,APTT,log2_EASIX, Los_ ICU and the use of vasopressor are independent risk factors of 28-day mortality of ARDS patients. In the CQMU cohort: Age, P/F, log2_EASIX, Los_ ICU and the use of vasopressor are independent risk factors of 28-day mortality of ARDS patients. Abbreviations:Los_ ICU, ICU length of stay (day); SOFA, sequential organ failure assessment; EASIX, endothelial activation and stress index; P/F, ratio of arterial oxygen partial pressure to inspired oxygen concentration; BUN, blood urea nitrogen (mg/dL); INR, international normalized ratio; PT, prothrombin time(s); APTT, activated partial thromboplastin time(s); ALT, Alanine aminotransferase (U/L); AST, Aspartate aminotransferase (U/L),Significance level <0.05.

The univariate Cox regression analysis of the 28-day all-cause mortality risk in both cohorts is illustrated in Supplementary Figure S1. Following the results from the univariate logistic regression analysis, a multivariable Cox regression analysis was conducted. In this multivariable Cox analysis, an increase in log2_EASIX levels was found to be significantly associated with the risk of 28-day all-cause mortality [MIMIC-IV cohort: HR = 1.216, 95% CI: 1.043–1.417, p = 0.013; CQMU cohort: HR = 1.448, 95% CI: 1.171–1.792, p = 0.001]. This finding suggests that elevated log2_EASIX levels may serve as a potential marker for assessing mortality risk in these populations. Furthermore, the use of vasopressors, a low oxygenation index, and a shorter ICU stay were also associated with an increased risk of 28-day mortality. The results from the multivariate Cox analysis are presented in Figure 2.

Furthermore, we enhanced the prediction model and established that the relationship between Log2_EASIX and the likelihood of all-cause mortality at 28 days remains statistically significant. This significance persists even after accounting for variables such as age, gender, length of ICU stay, and various comorbidities, including hypertension, chronic pulmonary conditions, diabetes, renal failure, and malignant cancer. Additionally, we considered treatments administered during intervention, such as vasopressors, renal replacement therapy, and invasive mechanical ventilation, as well as laboratory parameters including hemoglobin, WBC, BUN, P/F (PaO2/FiO2), SOFA, INR, PT, PTT, ALT, and AST (Table 2).

**TABLE 2:** Risk of 28-day Mortality according to Log2-EASIX in the MIMIC-IV cohort and CQMU cohort.

Variables	The MIMIC-IV cohort		The CQMU cohort	
	P Value	HR (95%CI)	P Value	HR (95%CI)
Non-adjusted	0.013	1.142 (1.049–1.245)	<0.0001	1.142 (1.291–1.736)
Model 1	<0.0001	1.171 (1.072–1.280)	<0.0001	1.626 (1.395–1.895)
Model 2	0.001	1.173 (1.069–1.286)	<0.0001	1.619 (1.386–1.890)
Model 3	<0.0001	1.206 (1.100–1.323)	<0.0001	1.553 (1.330–1.813)
Model 4	<0.0001	1.210 (1.091–1.341)	<0.0001	1.452 (1.244–1.695)
Model 5	0.016	1.219 (1.037–1.432)	<0.0001	1.454 (1.160–1.823)

Model 1 adjusted for age, gender.

Model 2 adjusted for model 1 plus comorbidities (Hypertension, Chronic pulmonary disease, Diabetes, Renal failure, and Malignant cancer).

Model 3 adjusted for model 2 plus Los_ ICU.

Model 4 adjusted for model 3 plus treatments of intervention (Vasopressor + Continuous renal replacement treatment + Invasive mechanical ventilation).

Model 5 adjusted for model 4 plus hemoglobin, WBC, BUN, P/F(PaO2/FiO2), SOFA, INR, PT, PTT, ALT, AST.

**FIGURE 3:**
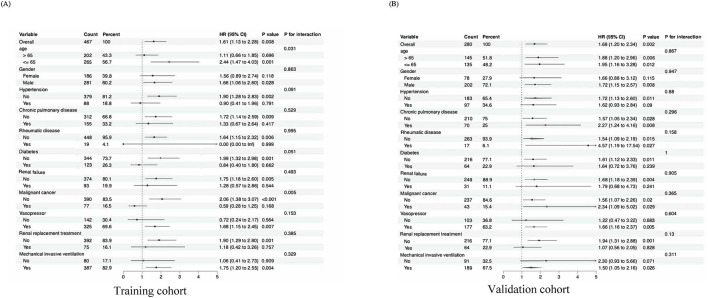
Subgroup analyses for the association of EASIX with 28-day mortality. **(A)** Forest plot for subgroup analysis of the association between EASIX (EASIX ≤ 1.48 vs EASIX>1.48) and 28-day mortality in ARDS patients for the MIMIC-IV cohort. **(B)** Forest plot for subgroup analysis of the association between EASIX (EASIX ≤ 2.10 vs EASIX>2.10) and 28-day mortality in ARDS patients for the CQMU cohort. Significance level <0.05.

Subgroup analyses were conducted to evaluate the consistency of the association between Log2_EASIX and 28-day mortality. The results indicated that no significant interactions were identified within any of the subgroups (interaction P > 0.05) across both cohorts. These subgroups included variables such as age, sex, comorbid conditions (hypertension, chronic pulmonary disorder, rheumatic conditions, diabetes, renal insufficiency, and cancer), as well as treatments such as CRRT, invasive mechanical ventilation, and vasopressor use (Figure 3). Similarly, the CQMU cohort did not exhibit any significant interactions across all subgroups for either the 60-day or 90-day mortality rates (S2 and S3), which was consistent with the findings for the 28-day mortality rate (P for interaction >0.05).

**FIGURE 4:**
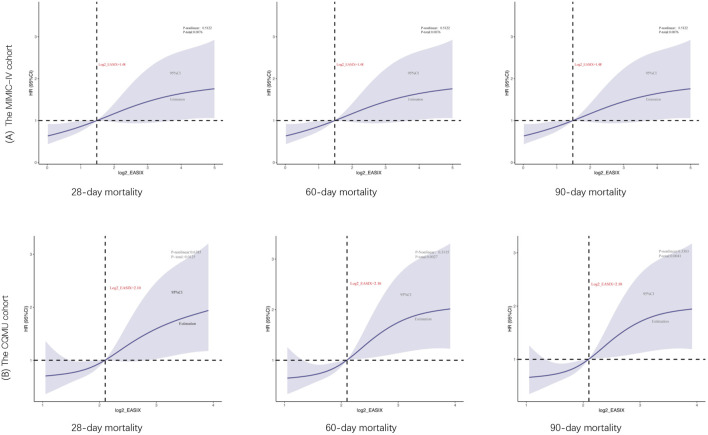
Restricted cubic spline curve for the log2_EASIX odds ratio (OR) for 28-day, 60-day, 90-day mortality. The MIMIC-IV cohort (cutoff value is 1.48) and the CQMU cohort (cutoff value is 2.10). The solid blue line represented the estimated ORs and the Solid blue curves represented corresponding 95% confidence intervals. The horizontal dashed black line and the black dot indicated the reference value. The p value for overall association <0.05 manifested a significant association, whatever the shape of the relationship curve was. The p value for non-linear association <0.05 indicated a nonmonotonic dose–response curve, otherwise, a monotonic relationship was suggested.

The truncated values derived from the receiver operating characteristic (ROC) curves were referenced to the restricted cubic spline (RCS) curves. In the RCS analysis, a statistically significant positive correlation was identified between increased log2_EASIX levels and the odds ratio (OR) for 28-day mortality, with a p-value of 0.0158. Within the MIMIC-IV cohort, a hazard ratio of 1.48 was determined, suggesting that higher log2_EASIX values are associated with an increased risk of mortality within this time frame. Consistently, the CQMU cohort with a threshold value of 2.10 further supported these findings, revealing a similar positive relationship between log2_EASIX levels and 28-day mortality rates. Notably, the analyses conducted across the MIMIC-IV and CQMU cohorts did not indicate significant nonlinearity in the relationship between log2_EASIX levels and mortality, as evidenced by p-values of 0.4390 and 0.6804, respectively; thus, a linear association was suggested. Additionally, similar results were observed in the context of 60-day and 90-day mortality, reinforcing the robustness of the findings across various follow-up durations (Figure 4).

**FIGURE 5:**
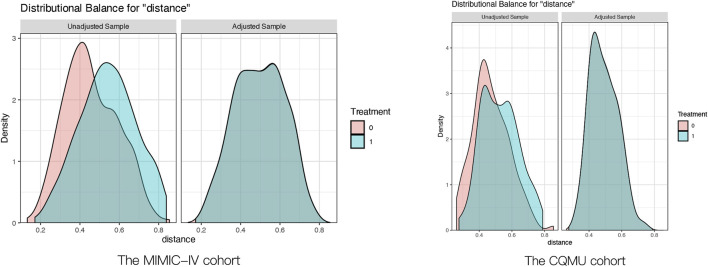
Pre‐ and post‐propensity score matching the difference of baseline characteristics between the two groups. The MIMIC-IV cohort:the Solid green curves represented Log2_EASIX ≤ 1.48, the Solid red curves represented Log2_EASIX>1.48; The CQMU cohort:the Solid green curves represented Log2_EASIX ≤ 2.10, the Solid red curves represented Log2_EASIX>2.10.

The cutoff values derived from the RCS curve for the MIMIC-IV cohort (Log2_EASIX = 1.48) and the CQMU cohort (Log2_EASIX = 2.10) were utilized to categorize the data into high Log2_EASIX and low Log2_EASIX groups. To ensure baseline balance, propensity score matching (PSM) was employed. Following PSM, the two groups demonstrated a satisfactory balance in baseline characteristics, as indicated in Supplementary Tables S2,S3. This balance post-PSM is further illustrated in Figure 5.

After propensity score matching (PSM), the analysis revealed that both before and after PSM, the higher Log2_EASIX group exhibited elevated levels of BUN, INR, PT, APTT, ALT, and AST compared to the lower Log2_EASIX group (Supplementary Tables S4,S5). This finding indicates that a higher Log2_EASIX may be associated with impaired liver and kidney function, as well as coagulation abnormalities. Furthermore, concerning patient prognosis and treatment interventions, both cohorts post-PSM demonstrated that the higher Log2_EASIX group experienced significantly increased 28-day mortality rates compared to the lower Log2_EASIX group [The MIMIC-IV cohort: 48 (34.3%) vs 27 (19.3%), p = 0.007; CQMU cohort: 51 (62.2%) vs 35 (42.7%), p = 0.019] (Tables 3,4). Additionally, the high Log2_EASIX group displayed a notable rise in 60-day and 90-day mortality rates, further corroborated after PSM (Tables 3,4).

**TABLE 3:** Impact of the level of Log2_EASIX on clinical outcomes in ARDS patients before and after propensity score matching in MIMIC-IV cohort.

Variables	Before PSM				After PSM			
	Log2_EASIX≤1.48	Log2_EASIX>1.48	p	SMD	Log2_EASIX≤1.48 (n = 140)	Log2_EASIX>1.48 (n = 140)	P	SMD
	(n = 234)	(n = 233)						
SOFA	7.00 [5.00, 9.00]	11.00 [9.00, 14.00]	<0.001	1.26	7.00 [5.00, 9.00]	11.00 [9.00, 14.00]	<0.001	1.245
Los-ICU	16.00 [10.75, 26.00]	14.00 [7.00, 21.25]	0.030	0.249	14.50 [9.00, 22.75]	14.50 [6.25, 21.00]	0.321	0.210
Vasopressor	148 (63.2%)	177 (76.0%)	0.004	0.279	88 (62.9%)	106 (75.7%)	0.028	0.281
CRRT	10 (4.3%)	65 (27.9%)	<0.001	0.679	6 (4.3%)	36 (25.7%)	<0.001	0.629
IMV	197 (84.2%)	190 (81.5%)	0.525	0.07	115 (82.1%)	117 (83.6%)	0.874	0.038
28d morbidity	41 (17.5%)	67 (28.8%)	0.006	0.269	27 (19.3%)	48 (34.3%)	0.007	0.344
60d morbidity	54 (23.1%)	75 (32.2%)	0.036	0.205	34 (24.3%)	52 (37.1%)	0.028	0.281
90d morbidity	59 (25.2%)	85 (36.5%)	0.011	0.246	39 (27.9%)	58 (41.4%)	0.024	0.288

Data are presented as the mean ± standard deviation (SD) for normal variables, median (IQR) for skewed variables, and numbers (proportions) for categorical variables. Standardized mean difference (SMD) > 0.1 suggests a potential imbalance, p-value <0.05 indicates a significant imbalance. Abbreviations:Los_ ICU, ICU, length of stay (day); SOFA, sequential organ failure assessment; EASIX, endothelial activation and stress index; HCRRT, Continuous renal replacement therapy (day); IMV, Invasive mechanical ventilation (day); PSM, propensity score matching; SMD, standardized mean differences.

**TABLE 4:** Impact of the level of Log2_EASIX on clinical outcomes in ARDS patients before and after propensity score matching in CQMU cohort.

Outcome	Before PSM				After PSM			
	Log2_EASIX≤2.10 (n = 140)	Log2_EASIX>2.10 (n = 140)	p	SMD	Log2_EASIX≤2.10	Log2_EASIX>2.10	p	SMD
					(n = 82)	(n = 82)		
SOFA	6.00 [4.00, 9.00]	7.00 [4.00, 11.00]	0.099	0.193	6.00 [4.00, 9.00]	6.00 [4.00, 10.00]	0.808	0.040
Los_ ICU	5.00 [3.00, 10.00]	8.00 [4.00, 13.00]	<0.001	0.26	5.00 [3.00, 9.00]	7.00 [3.75, 12.00]	0.052	0.115
Vasopressor	85 (60.7%)	92 (65.7%)	0.457	0.104	48 (58.5%)	54 (65.9%)	0.421	0.151
CRRT	120 (85.7%)	96 (68.6%)	0.001	0.417	74 (90.2%)	59 (72.0%)	0.005	0.481
IMV	49 (35.0%)	41 (29.3%)	0.370	0.123	30 (36.6%)	24 (29.3%)	0.406	0.156
28d morbidity	53 (37.9%)	84 (60.0%)	<0.001	0.454	35 (42.7%)	51 (62.2%)	0.019	0.398
60d morbidity	59 (42.1%)	95 (67.9%)	<0.001	0.535	35 (42.7%)	57 (69.5%)	0.001	0.562
90d morbidity	65 (46.4%)	96 (68.6%)	<0.001	0.460	37 (45.1%)	58 (70.7%)	0.002	0.537

Data are presented as the mean ± standard deviation (SD) for normal variables, median (IQR) for skewed variables, and numbers (proportions) for categorical variables. Standardized mean difference (SMD) > 0.1 suggests a potential imbalance, p-value <0.05 indicates a significant imbalance. Abbreviations:Los_ ICU, ICU, length of stay (day); SOFA, sequential organ failure assessment; EASIX, endothelial activation and stress index; HCRRT, Continuous renal replacement therapy (day); IMV, Invasive mechanical ventilation (day); PSM, propensity score matching; SMD, standardized mean differences.

**FIGURE 6:**
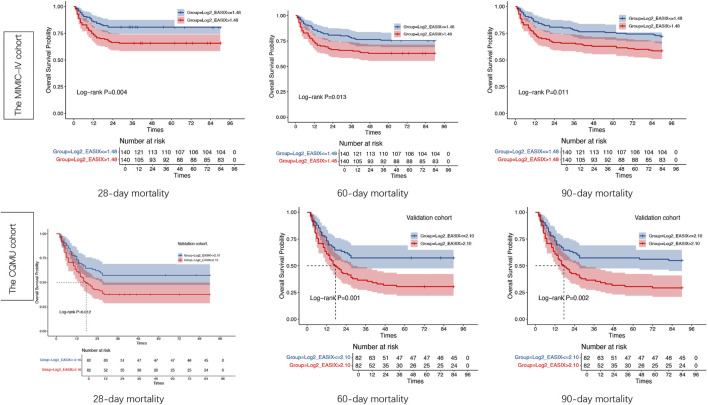
Kaplan–Meier survival analysis curves of the two cohorts. Kaplan–Meier curves showing the cumulative probability of all-cause mortality according to groups within 28, 60,90 days for the Medical Information Mart for Intensive Care-IV (MIMIC-IV) cohort and the CQMU cohort. EASIX index: in the MIMIC-IV cohort: Tertile red ( ≤ 1.48), Tertile blue (>1.48); n the CQMU cohort: Tertile red ( ≤ 2.10), Tertile blue (>2.10). Significance level <0.05.

The results of the Kaplan–Meier survival analyses for the two cohorts of post-PSM over 28 days, 60 days, 90 days are illustrated in Figure 6. The survival probabilities of the two cohorts are significantly different (p < 0.05). In both cohorts, the survival of the high log2_EASIX group is significantly lower than the lower one, and the difference among the two groups in the CQMU cohort is more significant than that in the MIMIC-IV cohort (Figure 6).

## Discussion

Endothelial cells constitute one of the largest organ systems in the human body, and their dysfunction can lead to coagulation complications, impaired vascular homeostasis, oxidative stress, and multi-organ damage. The pathophysiological process of Acute Respiratory Distress Syndrome (ARDS) involves the activation of endothelial cells and is associated with the systemic inflammatory response syndrome resulting from the disruption of vascular barrier integrity (Vassiliou et al., 2020). Lactate dehydrogenase, platelet counts, and creatinine levels have been shown to be markers of endothelial dysfunction (Cai et al., 2023; Millar et al., 2016). A study conducted by Henry indicated that a key indicator of severe COVID-19 infection progressing to ARDS is the direct secretion of serum LDH triggered by endothelial damage caused by ECs and circulating cells, including platelets and leukocytes (Bye et al., 2021). Additionally, elevated creatinine levels are associated with endothelial dysfunction and renal impairment (Xu et al., 2023). Another study has shown that platelets and fibrinogen work together to mediate endothelial damage through multiple signaling pathways, which is believed to be an important contributor to the pathogenesis of critical ARDS (Barrett et al., 2021). A different study found that Mitofusin-2 (Mfn2) in megakaryocytes and platelets can maintain the mitochondrial phenotype, and there is a significant correlation with the 28-day mortality rate of patients with ALI (Henry et al., 2020). In LPS-induced ALI, glycoprotein VI (GPVI)-mediated platelet activation significantly contributes to neutrophil migration, the formation of platelet-neutrophil complexes, and neutrophil activation. Additionally, administering anti-GPVI therapy enhanced the effectiveness of the alveolar-capillary barrier and mitigated physiological damage in mice subjected to LPS treatment (Dixon et al., 2012). These findings suggest that the measurement of these three components may have synergistic effects in reflecting vascular endothelial activation and injury. Therefore, based on the role of endothelial injury in the pathogenesis of ARDS, it is reasonable to assume that EASIX is a promising prognostic parameter in patients with the condition.

Previous research on EASIX levels has primarily concentrated on allogeneic hematopoietic stem cell transplantation. Notably, EASIX levels measured before the preconditioning phase have demonstrated an ability to predict both overall survival rates and relapse-free survival rates following allogeneic hematopoietic stem cell transplantation (allo-SCT) (Jacob et al., 2024). Additionally, studies have demonstrated that EASIX levels evaluated 1 year after the allo-SCT procedure can serve as indicators for identifying high-risk patients who may experience late non-relapse mortality (Burkard et al., 2023). Nevertheless, to the best of our knowledge, there is currently no study that has explored the impact of EASIX on mortality in patients with ARDS. This study aims to address this gap, COVID-19, coronary heart disease, small cell lung cancer, among others (Luft et al., 2021; Xu et al., 2023; Burkard et al., 2023; Kordelas et al., 2023; Finke et al., 2024). In this study, two distinct cohorts were utilized to demonstrate that the index-EASIX serves as a significant marker for assessing risk in patients with ARDS. The MIMIC database, which includes many patients hospitalized in the ICU, was employed as the MIMIC-IV cohort, while data from the ICU of the Second Affiliated Hospital of Chongqing Medical University served as the CQMU cohort. This approach was taken to validate the connection between EASIX and the prognosis of patients with ARDS. Our findings confirm the relationship between elevated EASIX levels and higher mortality rates in patients with ARDS across various ethnic groups and healthcare environments, as evidenced by consistent outcomes from both cohorts.

To our knowledge, this is the first study to investigate the correlation between the prognostic outcomes of patients with ARDS and EASIX levels. Our findings indicate a significant correlation between EASIX levels and 28-day mortality in ARDS patients, which was further validated in the CQMUcohort. EASIX serves as a simple and easily interpretable indicator, making it a valuable parameter for risk stratification. We observed that the higher log2_EASIX group exhibited significantly elevated mortality rates at 28 days, 60 days, and 90 days compared to the lower group. This result remained consistent across various subgroups, both before and after propensity score matching (PSM) in both cohorts. However, we did not find a significant relationship between log2_EASIX and the oxygenation index or the use of invasive mechanical ventilation, which may be partly attributed to the small sample size. Furthermore, while it is well established that respiratory oxygenation is closely linked to the functionality of the blood-gas barrier, EASIX primarily assesses vascular endothelial damage, leaving its effect on alveolar epithelium unclear. Therefore, we hypothesize that combining log2_EASIX with other markers of alveolar epithelial injury could potentially enhance its predictive capacity regarding respiratory oxygenation and the necessity for invasive mechanical ventilation. In our study, the results of log2_EASIX were inconsistent between the two cohorts concerning Renal Replacement Therapy and the use of vasopressors, which may be related to differences in sample size, underlying diseases, and the severity of patients’ conditions across the two cohorts.

Although there is a notable similarity in the correlation observed between EASIX and increased mortality rates among patients with ARDS, the RCS analysis reveals a linear correlation between log2_EASIX and the mortality rate across the two cohorts studied. However, distinctions persist between the two cohorts. Several factors may contribute to these differences, and these factors are as follows: Region-based differences in research institutions, including demographic factors (the MIMIC-IV cohort predominantly consists of Caucasians, whereas the CQMU cohort primarily comprises Asian patients) and differences in interventions (there is a higher percentage of invasive mechanical ventilation in the MIMIC-IV cohort, while the CQMU cohort displays a greater incidence of RRT). (Bos and Ware, 2022). The inconsistency in the severity of ARDS in the MIMIC-IV cohort: Compared with the MIMIC-IV cohort, the CQMU cohort has higher log2_EASIX values [2.10 (1.68–2.70) vs 1.48 (0.41–2.98), P < 0.01], and the CQMU cohort had worse oxygenation indices and longer ICU stays, which may indicate that patients in the CQMU cohort have more severe conditions than those in the MIMIC-IV cohort. However, the SOFA scores in the CQMU cohort were found to be lower than those in the MIMIC-IV cohort, which presents a paradox. We propose that this discrepancy may be attributed to two factors: 1) the MIMIC cohort included a higher proportion of patients with renal failure, potentially resulting in elevated SOFA scores; 2) the evaluation of central nervous system function in ARDS patients, especially those undergoing mechanical ventilation, is inherently challenging and subjective due to the administration of sedative medications (Moreno et al., 2023).

This study has multiple limitations that are noteworthy. Firstly, because our study employs a retrospective design, there remains the possibility of residual confounding, even after attempting to address potential confounding variables, such as age, sex, comorbidities, etc. Secondly, different pathophysiological factors—like the dysfunction or replacement of hematopoietic cells, along with liver and kidney functions—might affect the parameters necessary for EASIX calculation; therefore, additional validation through prospective studies is essential to verify EASIX’s role as a prognostic marker in individuals with ARDS. Ultimately, the absence of follow-up EASIX measurements throughout the ICU admission period restricted the evaluation of dynamic changes in EASIX and their effect on outcomes. These questions all enormously have limited it in the clinical on application. Hence, additional research is required to further explore the possible association between EASIX levels and mortality in patients with ARDS, as well as to investigate how EASIX fluctuations might influence patient outcomes.

## Conclusion

Elevated levels of EASIX were found to be positively correlated with a higher risk of all-cause mortality within 28, 60, and 90 days among patients with ARDS. Assessing EASIX levels could enhance the application of effective disease management strategies and offer important insights for clinical decision-making.

## Data Availability

The raw data supporting the conclusions of this article will be made available by the authors, without undue reservation.

## Glossary

EASIX: Endothelial Activation and Stress Index
ARDS: Acute Respiratory Distress Syndrome
LDH: Lactate dehydrogenase
PLT: Platelet
MIMIC-IV: Medical Information Mart for Intensive Care-IV
ICU: Intensive Care Unit
ALI: Acute lung injury
miR-1: MicroRNA-1
EC: Endothelial cell
GvHD: Graft-versus-host disease
CQMU: Chongqing medical university
DUA: Data use agreement
WBC: White blood cells
Hb: Hemoglobin
BUN: Blood urea nitrogen
PT: Prothrombin time
PTT: Partial thromboplastin time
INR: International normalized ratio
ALT: Alanine aminotransferase
AST: Aspartate aminotransferase
P/F: Ratio of arterial oxygen partial pressure to inspired oxygen concentration
SOFA: Sequential organ failure assessment
CRRT: Continuous renal replacement therapy
IMV: Invasive mechanical ventilation
SD: Standard deviation
IQR: Interquartile range
CI: Confidence interval
HR: Hazard ratio
ROC: Receiver operating characteristic
RCS: Restricted cubic spline
OR: Odds ratio
COVID-19: Corona Virus Disease 2019
GPVI: Glycoprotein VI
LPS: Lipopolysaccharide
Mfn2: Mitofusin-2
allo-SCT: Allogeneic hematopoietic stem cell transplantation
Los_ ICU: ICU length of stay
