# Supplementary material for: Between endothelial activation and stress index (EASIX) and mortality in acute respiratory distress syndrome (ARDS) patients: a multicenter retrospective study
Source: Front Physiol. 2025 Sep 26;16:1570988. doi: 10.3389/fphys.2025.1570988 (PMC12511030; doi:10.3389/fphys.2025.1570988)
Supplement: Supplementary file 1 [file Supplementaryfile1.docx]

S1 Univariate Cox analysis for overall survival according to groups within 28 days for the MIMIC-IV cohort and the CQMU cohort.

| **Variables** | **The MIMIC-IV cohort** | | | |  | **The CQMU cohort** | | | | |
| --- | --- | --- | --- | --- | --- | --- | --- | --- | --- | --- |
|  | **HR** | **HR（95L)** | **HR (95H)** | **P value** |  | **HR** | **HR (95L)** | | **HR (95H)** | **P value** |
| Age | 1.012 | 1 | 1.024 | 0.055 |  | 1.018 | | 1.007 | 1.029 | 0.002 |
| Gender (male) | 0.891 | 0.608 | 1.304 | 0.552 |  | 0.973 | | 0.671 | 1.410 | 0.884 |
| Hypertension | 1.355 | 0.866 | 2.119 | 0.183 |  | 1.155 | | 0.817 | 1.633 | 0.415 |
| Chronic pulmonary disease | 0.868 | 0.576 | 1.307 | 0.498 |  | 1.443 | | 0.998 | 2.085 | 0.051 |
| Diabetes | 0.974 | 0.633 | 1.498 | 0.905 |  | 0.7 | | 0.457 | 1.073 | 0.102 |
| Renal failure | 1.041 | 0.652 | 1.663 | 0.867 |  | 1.081 | | 0.658 | 1.774 | 0.759 |
| Malignant cancer | 1.383 | 0.872 | 2.192 | 0.168 |  | 1.19 | | 0.76 | 1.863 | 0.446 |
| Hemoglobin | 1.027 | 0.943 | 1.118 | 0.544 |  | 0.964 | | 0.91 | 1.021 | 0.211 |
| WBC | 1.008 | 0.98 | 1.036 | 0.583 |  | 1.008 | | 0.999 | 1.017 | 0.095 |
| BUN | 1.004 | 0.996 | 1.012 | 0.299 |  | 1.021 | | 1.004 | 1.039 | 0.018 |
| P/F | 0.995 | 0.992 | 0.999 | 0.005 |  | 0.994 | | 0.99 | 0.997 | <0.0001 |
| SOFA | 1.098 | 1.047 | 1.151 | <0.0001 |  | 1.058 | | 1.02 | 1.097 | 0.002 |
| INR | 1.314 | 1.146 | 1.505 | <0.0001 |  | 1.418 | | 1.018 | 1.973 | 0.039 |
| PT | 1.022 | 1.01 | 1.035 | <0.0001 |  | 1.036 | | 1.01 | 1.064 | 0.008 |
| APTT | 1.013 | 1.005 | 1.021 | 0.001 |  | 1.014 | | 1.001 | 1.026 | 0.033 |
| ALT | 1.000 | 1.000 | 1.000 | 0.087 |  | 1.000 | | 1.000 | 1.000 | 0.002 |
| AST | 1.000 | 1.000 | 1.000 | 0.187 |  | 1.001 | | 1.000 | 1.001 | 0.001 |
| Log2_EASIX | 1.142 | 1.049 | 1.245 | 0.002 |  | 1.497 | | 1.291 | 1.736 | <0.0001 |
| Vasopressor | 2.572 | 1.532 | 4.317 | <0.0001 |  | 3.263 | | 2.126 | 5.008 | <0.0001 |
| CRRT | 0.882 | 0.519 | 1.5 | 0.644 |  | 0.995 | | 0.67 | 1.479 | 0.981 |
| IMV | 1.341 | 0.777 | 2.313 | 0.292 |  | 3.1 | | 1.963 | 4.896 | <0.0001 |
| Los_ ICU | 0.926 | 0.89 | 0.963 | <0.0001 |  | 0.866 | | 0.841 | 0.892 | <0.0001 |

95L means the lower limit of the 95% confidence interval, 95U means the upper limit of the 95% confidence interval. Significance level <0.05.Abbreviations：Los_ ICU, ICU length of stay (day)；SOFA, sequential organ failure assessment; EASIX, endothelial activation and stress index; Hb, hemoglobin (g/L); WBC, white blood cell (K/L) ; P/F, ratio of arterial oxygen partial pressure to inspired oxygen concentration; BUN, blood urea nitrogen (mg/dL); INR, international normalized ratio; PT, prothrombin time(s); PTT, partial thromboplastin time(s); ALT, Alanine aminotransferase (U/L); AST, Aspartate aminotransferase(U/L); LDH, Lactate dehydrogenase(U/L); CRRT, Continuous renal replacement therapy(day); IMV, Invasive mechanical ventilation(day).HR, Hazard ratio.


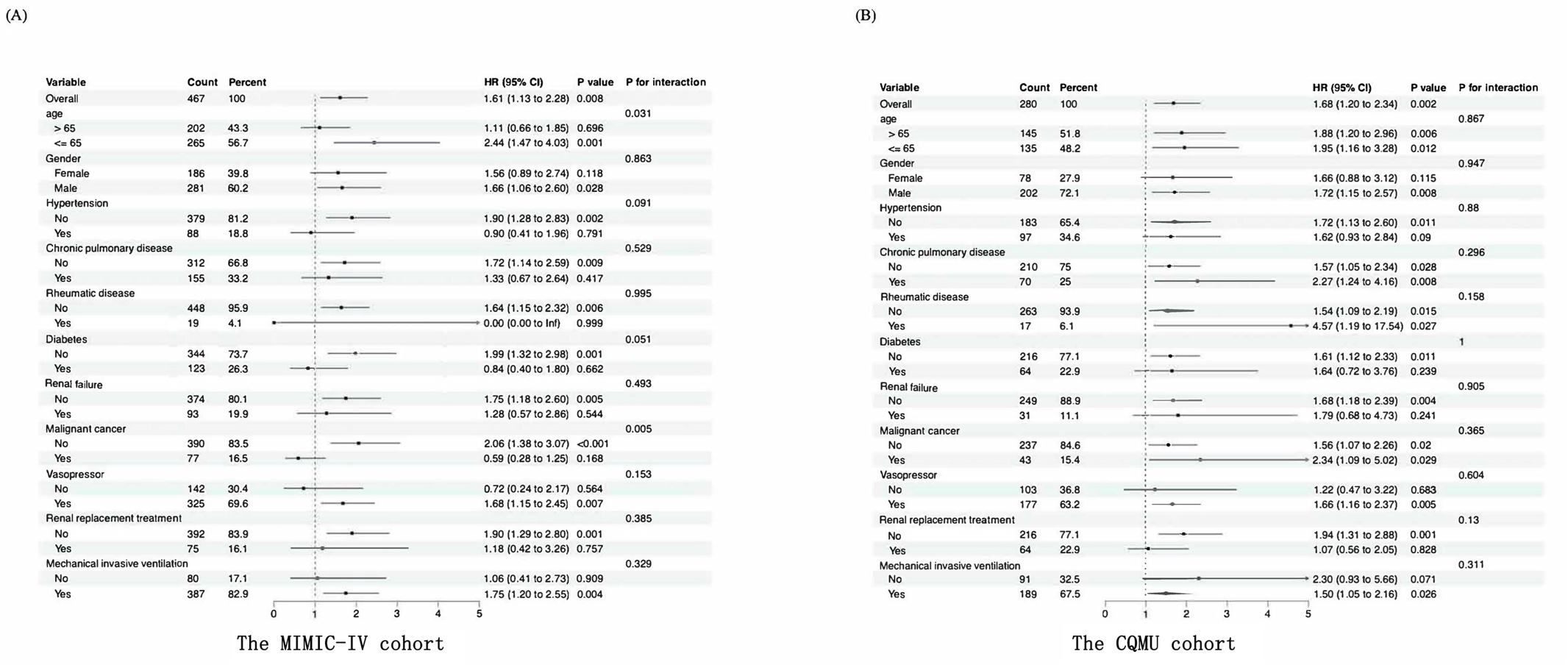


S2. **Subgroup analyses for the association of EASIX with 60-day mortality.** (A) Forest plot for subgroup analysis of the association between EASIX (EASIX<=1.48 vs. EASIX>1.48) and 60-day mortality in ARDS patients for the MIMIC-IV cohort. (B) Forest plot for subgroup analysis of the association between EASIX (EASIX<=2.10 vs. EASIX>2.10) and 60-day mortality in ARDS patients for the CQMU cohort. Significance level <0.05.


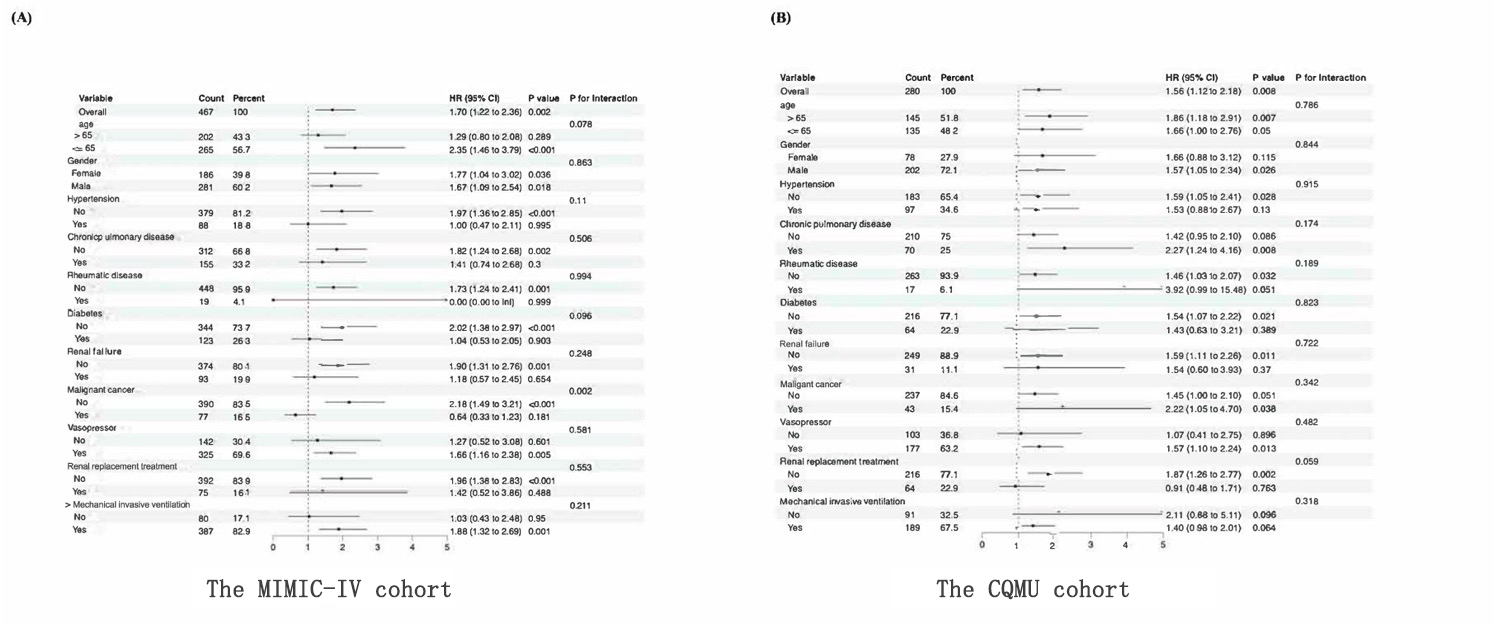


S3. **Subgroup analyses for the association of EASIX with 90-day mortality.** (A) Forest plot for subgroup analysis of the association between EASIX (EASIX<=1.48 vs. EASIX>1.48) and 90-day mortality in ARDS patients for the MIMIC-IV cohort. (B) Forest plot for subgroup analysis of the association between EASIX (EASIX<=2.10 vs. EASIX>2.10) and 90-day mortality in ARDS patients for the CQMU cohort. Significance level <0.05.

S4 Baseline characteristics of ARDS patient with absolute standardized mean differences pre‐ and post‐matching in MIMIC-IV cohort.

| **Variables** | **Before PSM** | | | |  | **After PSM** | | | |
| --- | --- | --- | --- | --- | --- | --- | --- | --- | --- |
|  | **Log2_EASIX≤1.48**  **(n=234)** | **Log2_EASIX>1.48**  **(n=233)** | **P value** | **SMD** |  | **Log2_EASIX≤1.48 (n=140)** | **Log2_EASIX>1.48 (n=140)** | **P value** | **SMD** |
| Age | 64.50 [53.00, 77.00] | 62.00 [50.00, 72.00] | 0.02 | 0.213 |  | 61.48 [50.25, 74.00] | 60.31[50.25, 70.75] | 0.54 | 0.073 |
| Gender (female) | 124 (53.0%) | 157 (67.4%) | 0.002 | 0.297 |  | 85 (60.7%) | 83 (59.3%) | 0.903 | 0.029 |
| Hypertension | 49 (20.9%) | 39 (16.7%) | 0.297 | 0.108 |  | 27 (19.3%) | 27 (19.3%) | 1 | <0.001 |
| Chronic pulmonary disease | 82 (35.0%) | 73 (31.3%) | 0.451 | 0.079 |  | 45 (32.1%) | 40 (28.6%) | 0.603 | 0.078 |
| Diabetes | 67 (28.6%) | 56 (24.0%) | 0.306 | 0.105 |  | 35 (25.0%) | 34 (24.3%) | 1 | 0.017 |
| Renal failure | 30 (12.8%) | 63 (27.0%) | <0.001 | 0.362 |  | 24 (17.1%) | 21 (15.0%) | 0.745 | 0.058 |
| Malignant cancer | 33 (14.1%) | 44 (18.9%) | 0.205 | 0.129 |  | 21 (15.0%) | 24 (17.1%) | 0.745 | 0.058 |

PSM, propensity score matching; SMD, standardized mean differences. Standardized mean difference (SMD) >0.1 suggests a potential imbalance, p-value < 0.05 indicates a significant imbalance.

S5 Baseline characteristics of ARDS patient with absolute standardized mean differences pre‐ and post‐matching in CQMU cohort.

| **Variables** | **Before PSM** | | | |  | **After PSM** | | | |
| --- | --- | --- | --- | --- | --- | --- | --- | --- | --- |
|  | **Log2_EASIX≤2.10（n=140）** | **Log2_EASIX>2.10（n=140）** | **P value** | **SMD** |  | **Log2_EASIX≤2.10**  **（n=82）** | **Log2_EASIX>2.10**  **（n=82）** | **P value** | **SMD** |
| Age | 67.00 [56.00, 77.00] | 63.00 [52.00, 74.00] | 0.016 | 0.334 |  | 66.00 [55.00, 74.75] | 66.00 [57.25, 74.00] | 0.858 | 0.032 |
| Gender (female) | 109 (77.9%) | 93 (66.4%) | 0.046 | 0.257 |  | 65 (79.3%) | 63 (76.8%) | 0.850 | 0.059 |
| Hypertension | 51 (36.4%) | 46 (32.9%) | 0.615 | 0.075 |  | 33 (40.2%) | 25 (30.5%) | 0.253 | 0.205 |
| Chronic pulmonary disease | 38 (27.1%) | 32 (22.9%) | 0.490 | 0.099 |  | 22 (26.8%) | 16 (19.5%) | 0.355 | 0.174 |
| Diabetes | 38 (27.1%) | 26 (18.6%) | 0.117 | 0.205 |  | 15 (18.3%) | 17 (20.7%) | 0.844 | 0.062 |
| Renal failure | 15 (10.7%) | 16 (11.4%) | 1.000 | 0.023 |  | 10 (12.2%) | 7 (8.5%) | 0.608 | 0.120 |
| Malignant cancer | 23 (16.4%) | 20 (14.3%) | 0.740 | 0.059 |  | 11 (13.4%) | 15 (18.3%) | 0.521 | 0.134 |

PSM, propensity score matching; SMD, standardized mean differences. Standardized mean difference (SMD) >0.1 suggests a potential imbalance, p-value < 0.05 indicates a significant imbalance.

S6 Laboratory results of ARDS patient with absolute standardized mean differences pre‐ and post‐matching in MIMIC-IV cohort.

| **Variables** | **Before PSM** | | | |  | **After PSM** | | | |
| --- | --- | --- | --- | --- | --- | --- | --- | --- | --- |
|  | **Log2_EASIX≤1.48**  **(n=234)** | **Log2_EASIX>1.48**  **(n=233)** | **P value** | **SMD** |  | **Log2_EASIX≤1.48 (n=140)** | **Log2_EASIX>1.48 (n=140)** | **P value** | **SMD** |
| Hemoglobin | 11.20 [9.60, 12.90] | 10.35 [8.85, 12.20] | 0.001 | 0.276 |  | 11.23[9.67, 12.90] | 10.61 [8.85, 12.28] | 0.019 | 0.283 |
| WBC | 12.75 [9.61, 17.41] | 11.90 [7.75, 18.00] | 0.062 | 0.115 |  | 12.68 [9.39, 17.24] | 11.93 [8.28, 18.34] | 0.343 | 0.043 |
| BUN | 19.00 [13.62, 27.38] | 32.00 [21.50, 51.50] | <0.001 | 0.817 |  | 19.00 [13.50, 26.62] | 30.50 [21.88, 49.62] | <0.001 | 0.756 |
| P/F | 201.28 [150.27, 242.38] | 196.25 [153.33, 242.50] | 0.576 | 0.05 |  | 193.23[143.21, 238.88] | 192.44 [154.70, 239.21] | 0.909 | 0.014 |
| INR | 1.20 [1.10, 1.50] | 1.45 [1.20, 1.90] | <0.001 | 0.337 |  | 1.20 [1.10, 1.50] | 1.45 [1.20, 1.90] | <0.001 | 0.447 |
| PT | 13.45 [12.15, 16.35] | 15.75 [13.40, 20.45] | <0.001 | 0.34 |  | 13.43 [12.14, 16.47] | 15.97 [13.10, 20.60] | <0.001 | 0.45 |
| APTT | 32.30 [27.81, 41.35] | 37.65 [30.95, 49.00] | <0.001 | 0.266 |  | 31.92 [27.60, 41.05] | 37.35 [31.11, 48.08] | <0.001 | 0.329 |
| ALT | 28.50 [17.00, 56.38] | 60.00 [26.00, 209.50] | <0.001 | 0.458 |  | 30.75 [17.00, 62.00] | 72.00 [25.75, 297.25] | <0.001 | 0.499 |
| AST | 41.00 [25.00, 83.00] | 117.50 [52.00, 435.00] | <0.001 | 0.522 |  | 41.50 [23.50, 94.38] | 157.50 [55.00, 500.62] | <0.001 | 0.553 |

PSM, propensity score matching; SMD, standardized mean differences. Standardized mean difference (SMD) >0.1 suggests a potential imbalance, p-value < 0.05 indicates a significant imbalance. Hb, hemoglobin (g/L); WBC, white blood cell (K/L) ; BUN, blood urea nitrogen (mg/dL); P/F, ratio of arterial oxygen partial pressure to inspired oxygen concentration; INR, international normalized ratio; PT, prothrombin time(s); APTT, activated partial thromboplastin time(s);ALT, Alanine aminotransferase (U/L); AST, Aspartate aminotransferase(U/L).

S7 Laboratory results of ARDS patient with absolute standardized mean differences pre‐ and post‐matching in CQMU cohort.

| **Variables** | **Before PSM** | | | |  | **After PSM** | | | |
| --- | --- | --- | --- | --- | --- | --- | --- | --- | --- |
|  | **Log2_EASIX≤2.10（n=140）** | **Log2_EASIX>2.10（n=140）** | **P value** | **SMD** |  | **Log2_EASIX≤2.10**  **（n=82）** | **Log2_EASIX>2.10**  **（n=82）** | **P value** | **SMD** |
| Hemoglobin | 11.40 [9.00, 13.60] | 11.50 [9.30, 13.20] | 0.921 | 0.026 |  | 11.65 [9.53, 13.70] | 11.30 [9.50, 13.10] | 0.534 | 0.074 |
| WBC | 10.46 [6.57, 14.57] | 11.54 [8.16, 15.85] | 0.086 | 0.055 |  | 11.13 [7.73, 15.05] | 11.91 [7.31, 17.41] | 0.418 | 0.203 |
| BUN | 8.62 [5.89, 13.23] | 9.85 [6.28, 15.56] | 0.083 | 0.219 |  | 7.44 [5.76, 10.56] | 10.32 [6.96, 14.75] | 0.005 | 0.363 |
| P/F | 158.00 [120.25, 203.25] | 139.00 [108.75, 176.50] | 0.004 | 0.371 |  | 168.00 [118.75, 205.50] | 137.00 [110.75, 174.00] | 0.009 | 0.458 |
| INR | 1.13 [1.03, 1.24] | 1.16 [1.06, 1.35] | 0.034 | 0.242 |  | 1.12 [1.01, 1.24] | 1.18 [1.06, 1.40] | 0.011 | 0.364 |
| PT | 14.50 [13.57, 15.80] | 15.00 [13.88, 16.75] | 0.022 | 0.284 |  | 14.40 [13.40, 15.78] | 15.15 [14.00, 17.23] | 0.008 | 0.397 |
| APTT | 17.05 [15.90, 18.02] | 17.70 [16.50, 19.22] | 0.001 | 0.279 |  | 17.05 [16.02, 18.00] | 17.85 [16.50, 19.53] | 0.010 | 0.342 |
| ALT | 35.00 [21.75, 53.25] | 67.00 [36.75, 147.00] | <0.001 | 0.325 |  | 32.50 [21.25, 49.50] | 63.00 [32.25, 154.50] | <0.001 | 0.385 |
| AST | 28.00 [17.00, 44.25] | 43.50 [25.75, 92.25] | <0.001 | 0.421 |  | 27.00 [15.50, 43.50] | 38.50 [21.00, 92.75] | 0.002 | 0.391 |

PSM, propensity score matching; SMD, standardized mean differences. Standardized mean difference (SMD) >0.1 suggests a potential imbalance, p-value < 0.05 indicates a significant imbalance. Hb, hemoglobin (g/L); WBC, white blood cell (K/L) ; BUN, blood urea nitrogen (mg/dL); P/F, ratio of arterial oxygen partial pressure to inspired oxygen concentration; INR, international normalized ratio; PT, prothrombin time(s); APTT, activated partial thromboplastin time(s);ALT, Alanine aminotransferase (U/L); AST, Aspartate aminotransferase(U/L).
